# Comparison of Auditory Steady-State Responses With Conventional Audiometry in Older Adults

**DOI:** 10.3389/fneur.2022.924096

**Published:** 2022-07-04

**Authors:** Hadeel Y. Tarawneh, Hamid R. Sohrabi, Wilhelmina H. A. M. Mulders, Ralph N. Martins, Dona M. P. Jayakody

**Affiliations:** ^1^School of Human Sciences, The University of Western Australia, Perth, WA, Australia; ^2^Ear Science Institute Australia, Subiaco, WA, Australia; ^3^Centre for Healthy Ageing, College of Science, Health, Engineering and Education, Murdoch University, Perth, WA, Australia; ^4^School of Medical and Health Sciences, Edith Cowan University, Joondalup, WA, Australia; ^5^Department of Biomedical Sciences, Faculty of Medicine and Health Sciences, Macquarie University, Sydney, NSW, Australia; ^6^Ear Science Centre, School of Surgery, The University of Western Australia, Perth, WA, Australia

**Keywords:** auditory steady-state response (ASSR), pure-tone audiometry (PTA), hearing, older adult, objective audiometry

## Abstract

Behavioral measures, such as pure-tone audiometry (PTA), are commonly used to determine hearing thresholds, however, PTA does not always provide reliable hearing information in difficult to test individuals. Therefore, objective measures of hearing sensitivity that require little-to-no active participation from an individual are needed to facilitate the detection and treatment of hearing loss in difficult to test people. Investigation of the reliability of the auditory steady-state response (ASSR) for measuring hearing thresholds in older adults is limited. This study aimed to investigate if ASSR can be a reliable, objective measure of frequency specific hearing thresholds in older adults. Hearing thresholds were tested at 500 Hz, 1000 Hz, 2000 Hz, and 4000 Hz in 50 participants aged between 60 and 85 years old, using automated PTA and ASSR. Hearing thresholds obtained from PTA and ASSR were found to be significantly correlated (p < .001) in a cohort consisting of participants with normal hearing or mild hearing loss. ASSR thresholds were significantly higher as compared to PTA thresholds, but for the majority of cases the difference remained within the clinically acceptable range (15 dB). This study provides some evidence to suggest that ASSR can be a valuable tool for estimating objective frequency-specific hearing thresholds in older adults and indicate that ASSR could be useful in creating hearing treatment plans for older adults who are unable to complete behavioral PTA. Further research on older adults is required to improve the methodological features of ASSR to increase consistency and reliability, as well as minimize some of the limitations associated with this technique.

## Introduction

Sensory processing declines across adulthood, with one third of those over the age of 65 being affected by disabling hearing loss (HL) (1). It is estimated that around 466 million people worldwide have disabling HL, accounting for over 5% of the world's population (1). The World Health Organization estimates that untreated hearing loss has an annual global cost of approximately US$750 billion. The identification of and addressing HL can be cost-effective and beneficial at an individual and societal level. Hearing loss can have great impact on quality of daily living and on communication (2–4). In addition, untreated HL is associated with multiple co-morbidities, including anxiety (5), depression (6), social isolation (7), loneliness (8) and poor physical health (9).

Assessing auditory acuity is frequently obtained using pure-tone audiometry (PTA), the gold standard for evaluating hearing threshold status, however, PTA does not always provide reliable hearing information in difficult to test individuals (10). This can be due to lack of cooperation during the assessment, inability to maintain attention and focus or limited understanding of test instructions (11). Older adults with cognitive impairment, particularly at moderate-to-severe levels, can have difficulties in performing behavioral hearing assessments, due to their diminished ability to maintain attention and understand test instructions (12). However, detecting and treating HL in those with cognitive impairment can have positive implications on their cognitive performance (13–16), social interaction (15) and overall quality of life (2). Therefore, being able to objectively measure hearing function in older adults who are unable to complete behavioral PTA is of great interest.

The auditory brainstem response (ABR), a far-field auditory electrophysiological test conducted using surface electrodes, provides an objective alternative method for measuring hearing function and is used particularly in infants and children not suited for behavioral PTA. ABR includes auditory evoked potentials from the eighth cranial nerve (auditory nerve) and neurons along the brainstem auditory pathway after presentation of an acoustic stimulus (17). ABR evoked using click stimuli provides a high degree of information regarding the integrity of the central and peripheral auditory pathways, particularly due to the reproducibility and stability of the waveform (18, 19).

However, a major limitation of the click-evoked ABR for assessing hearing sensitivity is its inability to determine frequency-specific hearing thresholds (20). As click-evoked ABR collects whole basilar membrane responses, it is difficult to accurately determine participating frequency ranges, which limits its effectiveness in providing accurate information for hearing loss intervention and rehabilitation. Commonly, ABR recordings are also dependent on the subjective interpretation of a recorded waveform by the examiner in order to evaluate if a response is present or not, and therefore, ABR results can be influenced by the examiner's experience and expertise (21, 22). Additionally, research has suggested that ABR testing cannot be used to evaluate severe-to-profound hearing loss, as it provides inadequate measures at thresholds >90 dB eHL (23, 24).

Tone-evoked (tone-burst) ABR can be used to assess responses in one ear to one frequency at a time, however, this is time consuming and, like click-evoked ABR, does not provide responses in cases of severe and profound hearing loss (21). Recently, a new ABR testing paradigm, parallel ABR (pABR), has been proposed to provide frequency-specific hearing threshold measures for multiple octave frequencies in both ears simultaneously (25). This new ABR technique uses independently randomized sequences of tone-burst stimuli to acquire ABR waveforms. pABR has been suggested to acquire waveforms with similar morphology of traditional ABR in a fraction of the recording time (25, 26). However, pABR technique still requires examiners to subjectively interpret recorded waveforms and its performance in assessing participants with HL or from different age groups has not been established yet.

Auditory steady-state responses (ASSR) has been suggested to be another objective audiometry test that can overcome some of the limitations associated with ABR (27). Similar to ABR, ASSR is a scalp-recorded auditory evoked potential (28). ASSR is a periodic electrical response evoked by periodically modulated tones, which is used to assess hearing sensitivity in patients of all ages and various degrees of sensorineural hearing loss without the need for patient participation (29, 30). Unlike ABR, which is evoked by a short stimuli at a relatively low repetition rate, ASSR is evoked using repeated pure tones at high repetition rates. ASSR uses amplitudes and phases in a spectral domain and is dependent on peak detection across a spectrum, meaning that the response is periodic and phase-locked to a modulation envelope (28). ASSR can be detected using frequency, time or spectral based analyses (28, 31). The neural generators of ASSR are dependent on the modulation frequencies used in the testing. Higher cortical and subcortical structures are suggested to generate responses to slower modulation rates (<50 Hz), while the auditory nerve and brainstem are suggested to respond to faster modulation rates (>80 Hz) (32, 33).

ASSR can be used to evaluate hearing sensitivity at a range of frequencies similar to behavioral PTA, using simultaneous stimulation and evaluation of multiple frequencies binaurally (33). ASSR results are presented as an electrophysiological audiogram, allowing for easy interpretation of hearing quality and for the preparation of medical reports (34). ASSR has also been suggested to provide better hearing data in comparison to ABR, in cases with severe-to-profound sensorineural hearing loss of 90 dB HL or greater (22, 35). Moreover, ASSR thresholds (spectrum of the response) are predicted by the stimulus spectrum and do not require subjective interpretations of the recorded responses, therefore overcoming some of the common limitations associated with other clinical audiometric tests, e.g., ABR.

Previous research suggests that ASSR can be a reliable predictor of hearing thresholds when compared to PTA in both children and adults (10, 11, 30, 35–37). However, there is no research comparing hearing threshold measures between PTA and ASSR in a cohort consisting of only older adults (aged 60 years and over), to date, research has only included older adults as part of a mixed aged (ranging from children to older adults) cohort when comparing PTA and ASSR thresholds (11, 30, 37). There is evidence to suggest that age-related changes in neural envelope processing and phase-locking may result in decreased ASSR responses in older adults compared to young adults or children (38–41). Therefore, the reliability of ASSR as a measure of hearing acuity specifically for older adults remains unclear. The aim of this study is to investigate if ASSR can be a reliable objective measure of frequency-specific hearing thresholds in older adults.

## Methods

### Participants

Community-dwelling (i.e., from the general population) older adults (aged 60 years and over) were recruited from an ongoing longitudinal research project known as the Western Australia Memory Study (WAMS). All procedures undertaken in this study were conducted in accordance with ethical approval (HPH-139) from the Ramsay Health Care WA| SA Human Research Ethics Committee (previously, the Hollywood Private Hospital Ethics Committee, Western Australia). As part of the WAMS, participants underwent comprehensive neuropsychological assessments, using self-reports and informant-reports questionnaires and surveys. All participants completed a demographic questionnaire and provided informed consent. Participants with current or previous diagnosis of a neurodegenerative disease, stroke or psychotic disorders were excluded from this study. Only older adults who performed within the normal range on cognitive measures were included in this study. More information on the neuropsychological and psychological assessments used in the WAMS can be found in Sohrabi et al. (42). Participants with unilateral deafness or already wearing hearing aids were excluded from this study. All participants underwent an otoscopic examination, a PTA and an ASSR, in the order given, in the same session/day. Only participants with normal otoscopic findings were included in the study.

### PTA Recording

Pure tone audiometry was conducted (air-conduction) bilaterally at 500, 1000, 2000, 4000, 8000 Hz using the KUDUwave 5000 system, Type 2 clinical audiometer (Emoyo, Johannesburg, South Africa). Tones were presented via insert earphones which were inserted in the ear canals with circumaural headphones placed over the ears. An automated threshold-seeking paradigm was used to establish hearing threshold. At each frequency, threshold levels were determined using the Hughson-Westlake (43) procedure, by increasing increments of 10 dB followed by decreasing increments of 5 dB. Participants were required to press a button in response to any tones they heard during the assessment. Degree of HL was classified based on the American Speech-Language-Hearing Association (ASHA) classification system adapted from Clark 1981 (44).

### ASSR Recording Parameters

ASSR was performed in an electrically shielded and sound attenuated room. Participants were tested while awake and in a relaxed Fowler's position (45). Air-conducted stimuli were presented to the left and right ear simultaneously via ER-3A insert earphones. Acoustic stimuli were generated and presented by the Chartr EP system (Version 5.3, GN Otometrics). Four carrier frequencies: 500, 1000, 2000, and 4000 Hz, were tested using an automated multiple ASSR technique that utilizes an algorithm that uses a Fourier Linear Combiner with an adaptive filter and circular statistical analysis (46). This means that the four carrier frequencies were tested simultaneously in both ears at each modulation frequency. 100% amplitude modulation and 20% frequency modulation were used for all carrier frequencies, with the response confidence set at 95% as predefined by the system manufacturer. The modulation frequency varied for each carrier frequency: modulation rates were 88, 80, 96, and 92 Hz for the right and 90, 82, 98, and 94 Hz for the left ear, for 500, 1000, 2000, and 4000 Hz carrier frequencies, respectively. A gain of 200 k, a low-pass filter at 105 Hz and a high-pass filter at 65 Hz were used.

ASSR recordings were obtained using four Ag/AgCl disc electrodes which were placed according to the International Electrode System (IES) 10-20; two inverting (reference) electrodes on each mastoid (one behind left ear and one behind right ear) behind the ear, non-inverting (active/recording) electrode at vertex (Cz) and ground electrode on the lower forehead. Prior to recording, the skin was prepared for electrode placement with a mild abrasive to obtain electrode impedances under 5 KΩ. ASSR measurements were performed using a descending procedure, by recording electrical responses while reducing the intensity of the acoustic signal in 10 dB steps until the threshold. The threshold was defined as the minimum intensity of detected responses, with a maximum of 7 min search time for each frequency allowed. Participants were not required to actively participate during ASSR recordings. Default correction factors (500 Hz−20 dB HL, 1000 Hz−10 dB HL, 2000 Hz−10 dB HL, 4000 Hz– 10 dB HL) on the Chartr EP system were applied to all final audiograms obtained from ASSR. To minimize artifacts and noise interference as a result of body movement, participants were instructed to stay still during the recording.

### Statistical Analysis

After the PTA and ASSR measurements, statistical analysis was performed using IBM SPSS Statistics, version 25.0 (IBM Corp, Armonk, NY). Continuous variables were presented as a mean with standard deviation, and categorical variables were presented as absolute numbers and percentages. A Student *t*-test was used to compare normally distributed continuous variables between PTA and ASSR measures. PTA and ASSR threshold measures were also compared with an assessment of the correlation using Pearson's correlation analysis. Frequencies in which ASSR testing did not elicit a response were excluded from the final statistical analysis. A *p*-value of < 0.05 was considered statistically significant.

## Results

A total of 50 (100 ears) community-dwelling older adults (14 male and 36 female) took part in this study (Table 1). Participants were aged between 61–84 years, average age for males was 72.9 ± 6 years and for females 71.6 ± 5.1 years (combined mean age 72.1 ± 5.4 years). On average, participant depression, anxiety, and stress scores were within normal levels (depression: 0–4, anxiety: 0–3, and stress: 0–7) according to the DASS 21 (47) severity scale: 2.2 ± 2.1, 2.2 ± 2.2 and 4.3 ± 2.9, respectively (Table 1). There was no significant correlation between psychological status (depression, anxiety, and stress) and participant age or gender (Pearson's correlation).

**Table 1:** Demographical characteristics of participants.

	**Sample (*n*)**	**Age (years)**	**Education (years)**	**MoCA score**	**Depression score**	**Anxiety score**	**Stress score**
Combined	50	72.1 ± 5.4	14.8 ± 2.5	26.9 ± 2.7	2.2 ± 2.1	2.2 ± 2.2	4.3 ± 2.9
Females	36	71.6 ± 5.1	14.9 ± 2.4	27.3 ± 2.9	2.1 ± 2	2 ± 1.9	4.5 ± 2.9
Males	14	72.9 ± 6	14.6 ± 2.7	26.2 ± 2.2	2.5 ± 2.3	2.6 ± 2.8	3.8 ± 2.8

### Behavioral PTA

Behavioral hearing thresholds using PTA were obtained for the whole sample. In this study, hearing range between 0–25 dB HL was considered normal hearing, 26–40 dB HL was considered mild HL, 41–55 dB HL was considered moderate HL, 56–70 dB HL was moderately severe HL, 71–90 was considered severe HL and 91 dB HL and above was considered profound HL. According to average 4-point PTA threshold measures, 78% (39/50) of participants had normal hearing thresholds (0–25 dB HL) and 22% (11/50) had mild hearing loss (26–40 dB HL). There was no significant correlation between PTA threshold measures and participants' depression, anxiety, or stress scores (Pearson's correlation). On average, males (24.6 dB ± 10.3, *n* = 14) had significantly higher hearing thresholds (*p* < 0.05) when compared to females (16.3 dB ± 7.2, *n* = 36), *t*_(48)_ = 3.26; *p* = 0.002. There was a low, however significant, correlation between PTA thresholds and participant age, *r*_(49)_ = 0.28; *p* < 0.05, showing increased thresholds with age.

### ASSR

ASSR thresholds could not be measured in 34%, 10%, 1% and 27% of ears for 500, 1000, 2000, and 4000 Hz frequencies, respectively. These cases were excluded from further statistical analysis for the frequency in which no response was measured; hence the number of data points differs between frequencies. ASSR testing took on average 20 min to complete, with the shortest time recorded to achieve threshold measures at all tested carrier frequencies being 3.5 min and the longest time being 30.5 min. Threshold measures for all four carrier frequencies were obtained in 36% (18/50) of participants. Of those participants the average 4-point hearing thresholds indicate 72.2% (13/18) had normal hearing, 22.2% (4/18) had mild hearing loss and 5.5% (1/18) had moderate hearing loss. There was no significant correlation between ASSR thresholds and participants' gender, depression, anxiety, and stress scores (Pearson's correlation). There was a moderate, and significant, linear positive correlation between ASSR thresholds and participant age *r*_(17)_ = 0.48; *p* < 0.05.

### Comparison of ASSR and PTA in Older Adults

**Figure 1:**
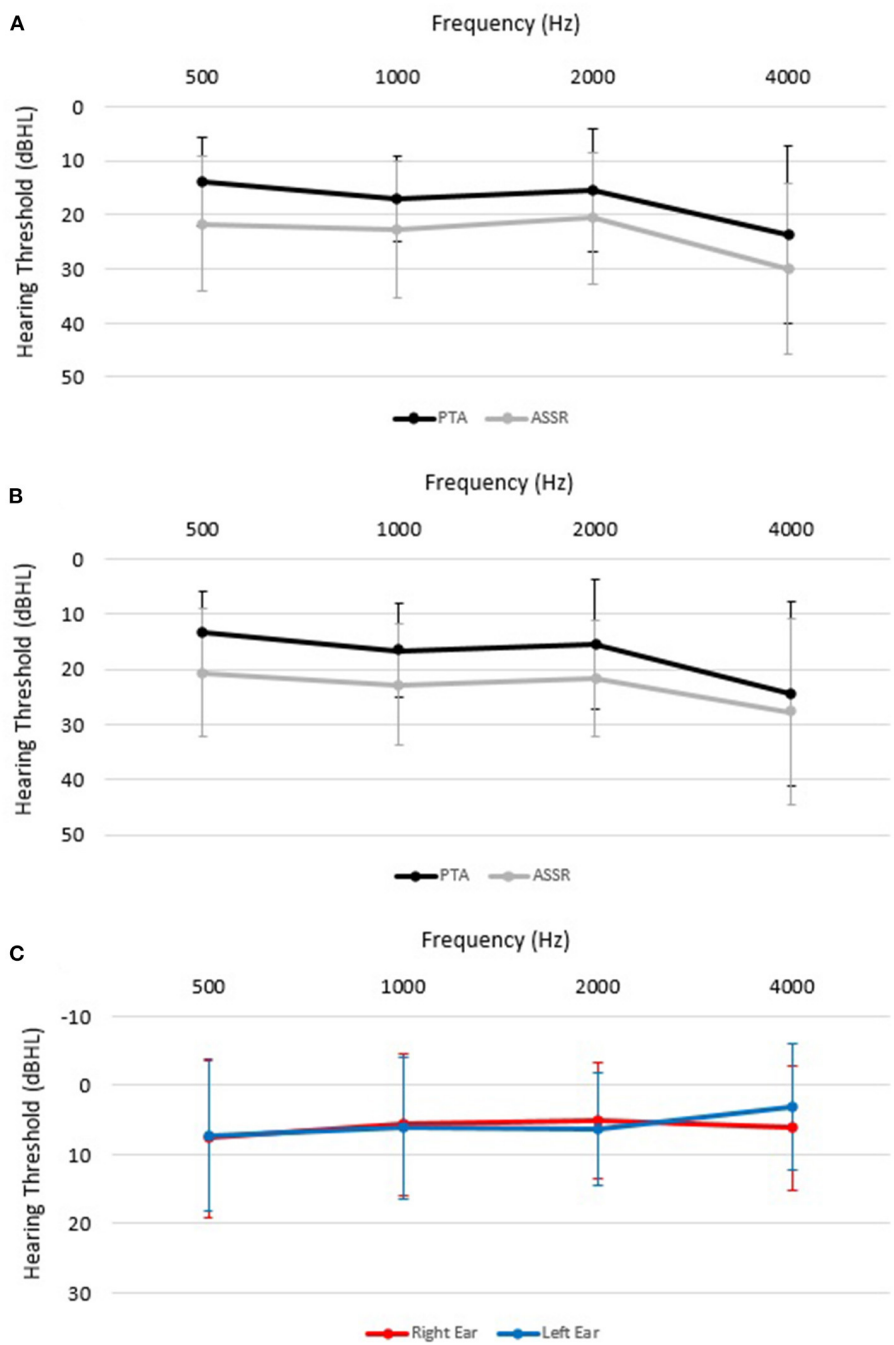
Mean (± SD) pure tone audiometry (PTA; black lines **A, B**) and auditory steady-state response (ASSR; gray lines **A, B**) hearing thresholds (in decibels dBHL) for each carrier frequency in the right ear **(A)**, left ear **(B)**. **(C)** shows threshold differences between PTA and ASSR in left and right ears.

Table 2 and Figure 1 provide a summary of the mean thresholds for each carrier frequency for both PTA and ASSR. ASSR thresholds were significantly higher as compared to PTA thresholds based on the paired sample *t*-test analyses. The significant difference between the two procedures (i.e., PTA and ASSR) was seen at all frequencies, 500 Hz (7.5 dB ± 11.2, *t*_(65)_ = 5.49; *p* < 0.001), 1000 Hz (6 dB ± 10.2, *t*_(89)_ = 5.58; *p* < 0.001), 2000 Hz (5.7 dB ± 8.2, *t*_(98)_ = 6.87; *p* < 0.001) and 4000 Hz (4.7 dB ± 9.2, *t*_(70)_ = 4.28; *p* < 0.001), in order from highest to lowest threshold difference (Table 2). Mean PTA and ASSR hearing threshold values and differences were similar for each carrier frequency when analyzed for left and right ears separately, as noted in Table 2 and Figure 1.

**Table 2:** Mean (± SD) pure tone audiometry (PTA) and auditory steady-state response (ASSR) hearing threshold values (in decibels dBHL) in normal hearing older adults.

**Frequency (Hz)**	**500**			**1000**			**2000**			**4000**		
**Ear**	**R (*n* = 34)**	**L** **(*n* = 32)**	**Combined (*n* = 66)**	**R** **(*n* = 45)**	**L (*n* = 45)**	**Combined** **(*n* = 90)**	**R (*n* = 50)**	**L** **(*n* = 49)**	**Combined (*n* = 99)**	**R** **(*n* = 35)**	**L (*n* = 36)**	**Combined** **(*n* = 71)**
PTA	13.9 ± 8.2	13.2 ± 7.2	13.6 ± 7.7	17.1 ± 8	16.6 ± 8.6	16.8 ± 8.2	15.5 ± 11.2	15.5 ± 11.7	15.5 ± 11.4	23.7 ± 16.3	24.5 ± 16.6	24.1 ± 16.3
ASSR	21.7 ± 12.4	20.6 ± 11.6	21.2 ± 11.9	22.8 ± 12.7	22.8 ± 11	22.8 ± 11.8	20.6 ± 12.1	21.8 ± 10.5	21.2 ± 11.3	30 ± 15.7	27.7 ± 16.7	28.8 ± 16.1
Difference	7.7 ± 11.6	7.3 ± 10.8	7.5 ± 11.2	5.7 ± 10.2	6.2 ± 10.2	6 ± 10.2	5.1 ± 8.3	6.3 ± 8.2	5.7 ± 8.2	6.2 ± 9.1	3.1 ± 9.2	4.7 ± 9.2
P-value	*P* <0.001	*P* <0.001	*P* <0.001	*P* <0.001	*P* <0.001	*P* <0.001	*P* <0.001	*P* <0.001	*P* <0.001	*P* <0.001	*P* = 0.046	*P* <0.001

*R, right ear, L, left ear*.

**Figure 2:**
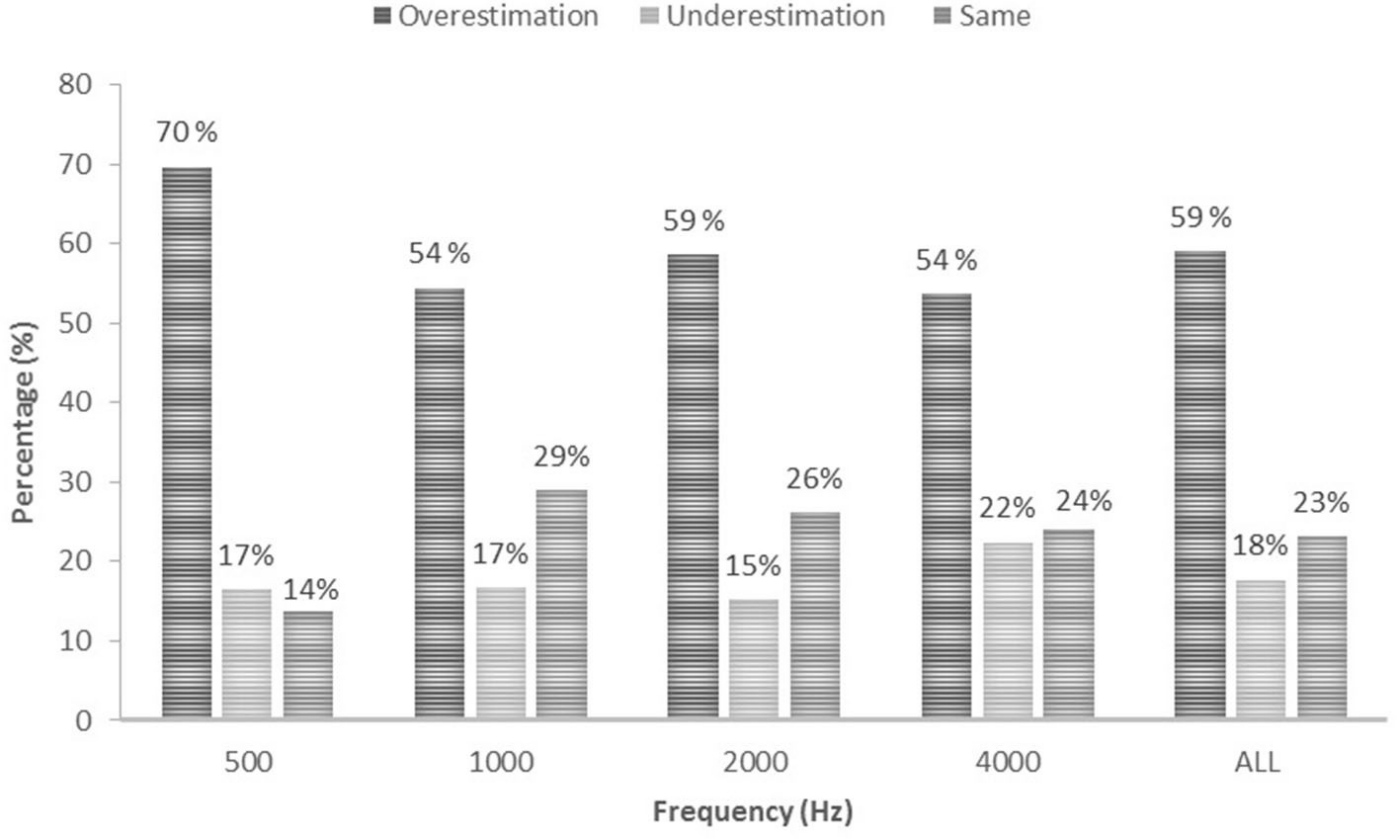
Accuracy of ASSR estimations in percentage (%) presented for each frequency and for all frequencies combined.

**Figure 3:**
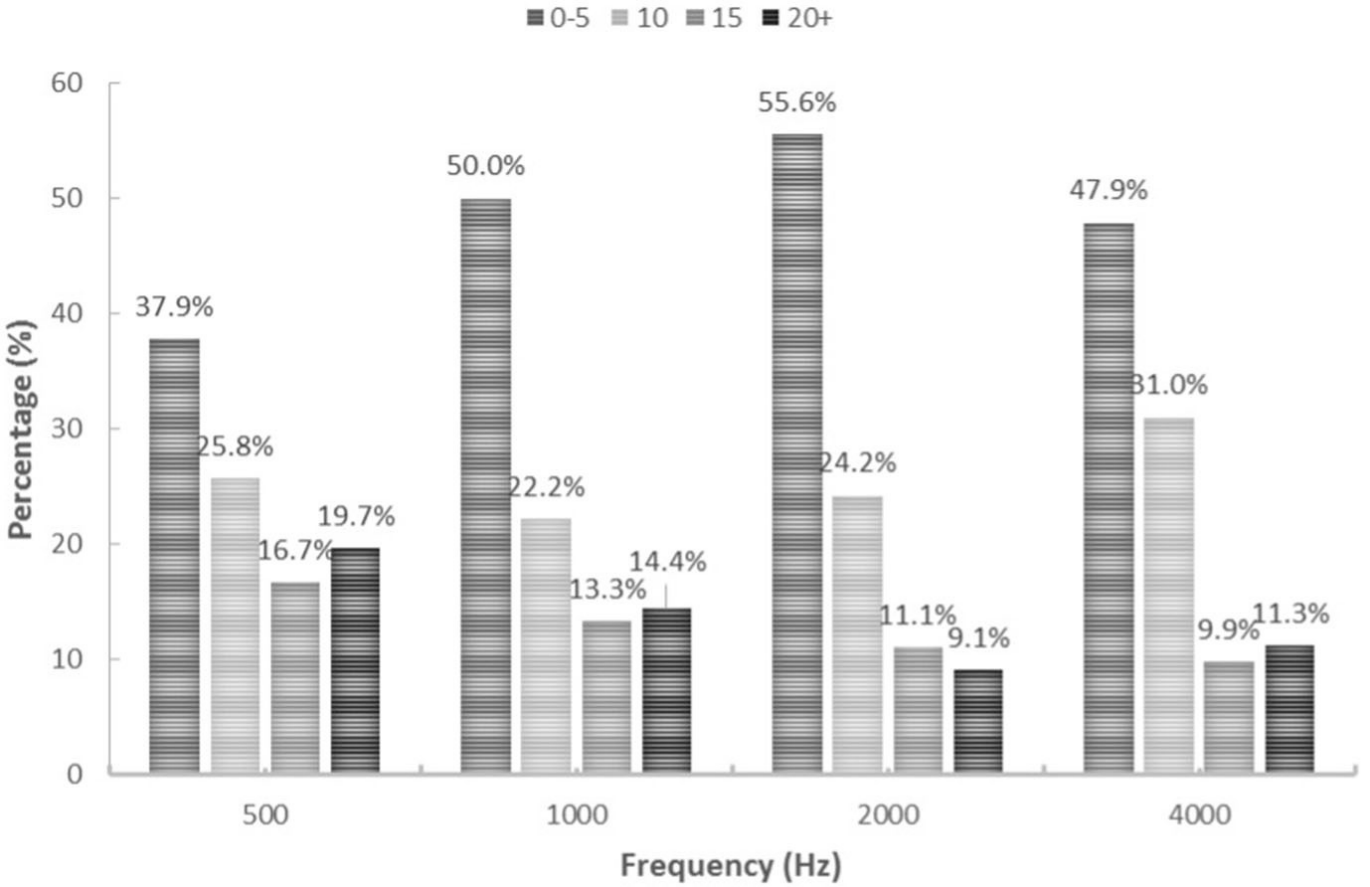
Distribution of ASSR and PTA differences for each frequency. Percentages presented for 0–5 dB, 10 dB, 15 dB and 20 dB+ (and over) threshold differences.

The majority of all thresholds measured using ASSR were higher than thresholds measured using PTA for the same ear. Overall, 59% of ASSR thresholds overestimated (were higher than) PTA thresholds, 18% underestimated PTA thresholds and 23% were the same as the PTA thresholds. A similar trend can be seen when looking at each carrier frequency separately, with the majority of ASSR thresholds overestimating the PTA threshold (Figure 2). Over 80% of hearing thresholds measured using ASSR were within ± 15 dB from thresholds measured using PTA at 500 (80.3%), 1000 (85.5%), 2000 (90.9%) and 4000 (88.7%) Hz. In total, 63.6% of thresholds measured using ASSR were within ±10 dB from PTA thresholds at 500 Hz, 72.2% at 1000 Hz, 79.8% at 2000 Hz and 78.9% at 4000 Hz. Distribution of ASSR and PTA threshold differences (dB HL) for each carrier frequency are presented in Figure 3.

**Figure 4:**
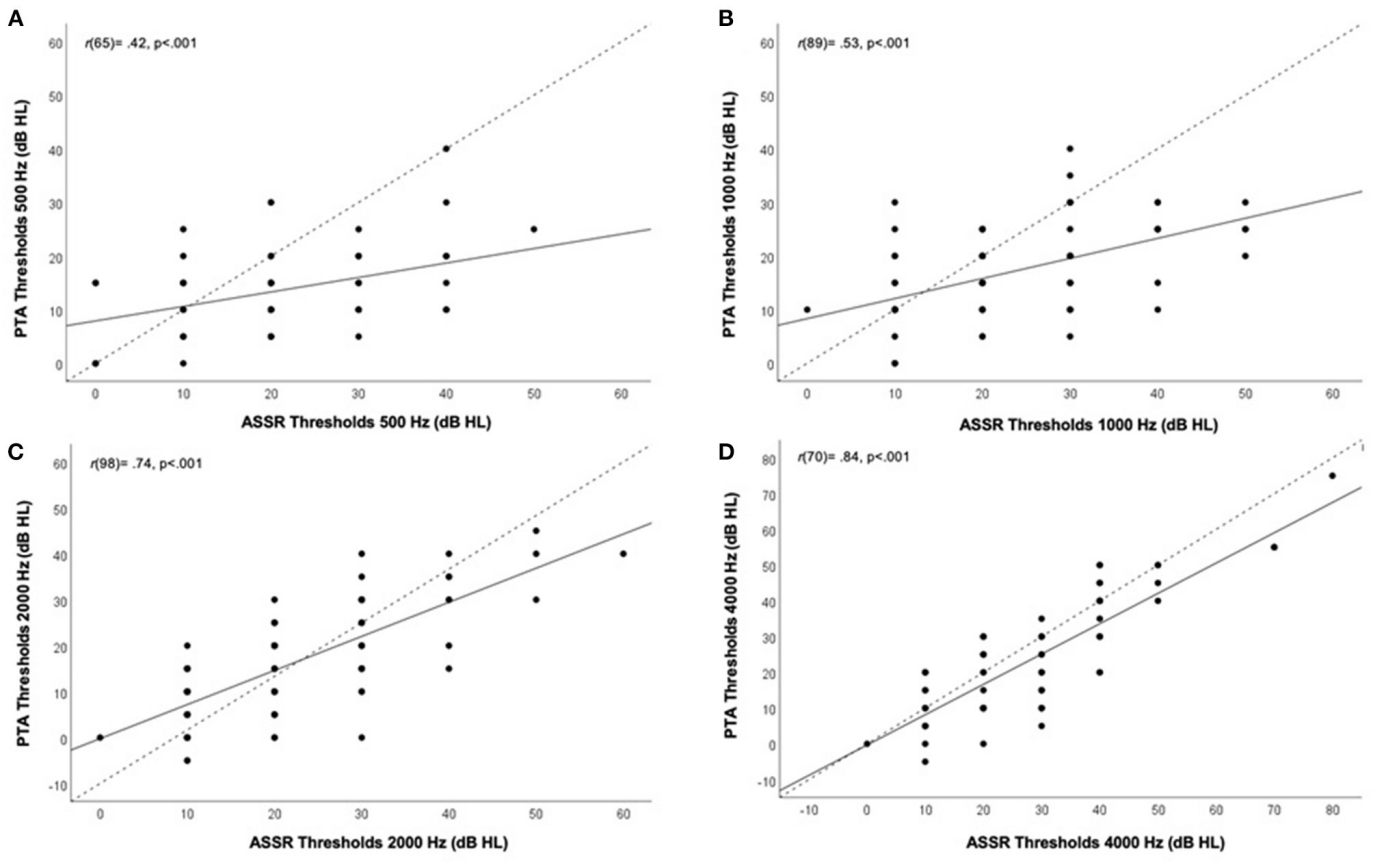
Correlation between hearing thresholds (both ears combined) obtained from auditory steady-state response (ASSR) (x-axis) and pure-tone audiometry (PTA) (y-axis) in dB HL according to carrier frequency. Different panels show different frequencies. **(A)** 500Hz, **(B)** 1000 Hz, **(C)** 2000 Hz, and **(D)** 4000 Hz. Correlation coefficients (r) and p-value are presented on the top left corner of each panel for all tested frequencies. Black line indicates line of best fit, dotted line indicates 1:1 ratio line.

Correlation analysis, as presented in Figure 4, revealed strong significant (*p* < 0.001) linear correlations between hearing threshold measures from ASSR and PTA at 1000 Hz, 2000 Hz and 4000 Hz at *r*_(89)_ = 0.53, *r*_(98)_ = 0.74, and *r*_(70)_ = 0.84, respectively. A moderate, yet significant, correlation between thresholds for ASSR and PTA was seen for the 500 Hz carrier frequency (*r*_(65)_ = 0.42; *p* < 0.001). Similarly, correlation analysis of each ear separately resulted in strong correlations for threshold measures between ASSR and PTA at 1000 Hz (right ear only), 2000 Hz and 4000 Hz and moderate correlations at 500 Hz and 1000 Hz (left ear only). See Supplementary Figures S1, S2 and Supplementary Table S1.

## Discussion

Hearing thresholds obtained from PTA and ASSR in the current study were found to be significantly correlated in a cohort consisting of elderly participants with normal hearing or mild hearing loss. This is in agreement with other studies that also reported a significant correlation between hearing thresholds obtained using PTA and ASSR (10, 37, 48). However, in the present study, there was a statistically significant increase in thresholds measured with ASSR as compared to PTA at all tested carrier frequencies. Mean threshold differences between PTA and ASSR were largest at 500 Hz with a difference of 7.5 dB HL and lowest at 4000 Hz with a difference of 4.7 dB HL. Nonetheless, we showed that the majority (varying between 64 and 79% dependent on carrier frequency) of threshold differences between ASSR and PTA were within 10 dB of each other and over 80% of ASSR thresholds were within 15 dB of PTA thresholds. Ten to fifteen dB differences in threshold measures are considered to be clinically acceptable and are tolerable when making hearing intervention plans (37, 49). Therefore, the results of this study indicate that threshold measures recorded using ASSR have the potential to provide useful objective estimations of hearing thresholds in “difficult to test” older adults for the timely detection and treatment of hearing loss. It should be noted that the present cohort of older adults presented with normal cognitive function and psychological status (i.e., depression, anxiety and stress), therefore, these factors would have no influence on test outcomes. Additionally, there was no statistical correlation between cognitive, psychological or gender status and hearing acuity.

In most cases, ASSR over estimated PTA thresholds. Over estimation of hearing thresholds could lead to an increased risk of false positives (identifying someone with HL even if hearing is normal) as well as over estimation of HL severity. Variations in the analysis algorithm and application of correction factors used to obtain ASSR and PTA thresholds could be contributing factors to the variation in threshold measures between the two techniques (49). In commercial acquisition systems, to counteract differences between ASSR and PTA, correction factors are applied based on the carrier frequency. Correction factors are set based on the difference between PTA and ASSR thresholds in subjects with varied hearing and age range (50). These correction factors can differ from one system manufacturer to another and are set as standard for most groups (i.e., adults, children, those with and without HL), which can result in variations in the threshold measures from one commercial system to another for the same subject (50). It may be that the correction factors applied to other age ranges may be suboptimal for older adults as used in the present study. Indeed, it has previously been suggested that ASSR can be a more useful technique in children, adults and older adults if the correction factors applied are defined specifically for each age group (50).

One factor that may influence the observed variations is the modulation rate used. In this study, for ASSR, default modulation rates were set for each carrier frequency according to manufacturer [Chartr EP (44)] recommendations. Modulation rates ranged from 80–98 Hz across the carrier frequencies. These modulation rates are considered fast modulations as they are over 50 Hz. Previous research that has informed acquisition system manufacturers, has been on participants in other age ranges [i.e., infants (35), adults under 35 (48), or a combination of children and adults (10, 11, 30, 36)] and this has yielded inconsistent ASSR protocols/recommendations. Previous research revealed that ASSRs are difficult to record in infants at low modulation frequencies (~40 Hz), therefore, high modulation frequencies have become a standard for ASSR testing regardless of the age of the subject (51). However, there is evidence to suggest that age-related changes in neural envelope processing may result in decreased ASSR responses for faster modulation rates in older adults compared to young adults or children (39, 40). Specifically, for high gamma frequencies (≥ 80 Hz) ASSRs decrease with age, which in turn suggests age-related decline in synchronized activity of high gamma oscillations (52–54). Therefore, this could contribute to the significant increase in thresholds seen when using ASSR compared to PTA in the present study.

Additionally, phase-locking of ASSR is suggested to be lower at high modulation rates in middle-aged and older adults in comparison to young adults (39). This has also been demonstrated by a number of animal studies, which show decline in phase-locking in fast modulations in both near- and far-field recordings with aging (55–57). The effect of aging on phase-locking is not reported for slow modulation frequencies (<50 Hz) (39). Reduced ASSR strength and lower phase-locking to fast modulation frequencies with aging is in line with reports of reduced temporal precision in encoding rapidly modulated stimuli as a result of loss of functional inhibition across the central auditory pathway with aging (38, 39, 41). Therefore, the use of high modulation rates for older adults may have resulted in no thresholds being established through ASSR in some cases and larger variations (less accuracy) in threshold differences between PTA and ASSR.

In this study ASSR thresholds were obtained for all tested carrier frequencies in only 36% of the participant sample (*n* = 50). Similarly, a study conducted on children (*n* = 20) found that ASSR thresholds were obtained for all frequencies tested (500, 1000, 2000, and 4000 Hz) in only 45% of the sample (35). Although, ASSR provides an objective measure of hearing thresholds without the need for active participation of the subject, it can be affected by patient movement, behavioral status (e.g., awake vs. asleep) and patient preparation (electrode impedance). Therefore, variations in these factors can result in inaccurate measures or no thresholds being established (11, 30). Additionally, a number of studies suggest that ASSR threshold measures at 500 Hz should be interpreted with caution (10, 30, 58). Similar to previous research, in the present study ASSR threshold measures at 500 Hz were the most variable among all carrier frequencies and also had the highest percentage of thresholds that were not established during the testing time limits (36, 59). This has been suggested to be due to the higher EEG noise and internal jittering as a result of neurologic asynchronicity (60). More research is required to establish strategies to overcome patient and equipment factors than may have negative impact on test results and accuracy.

### Study Limitations

One experimental protocol limitation that could have contributed to larger variations in threshold differences between ASSR and PTA is the difference in step sizes used for establishing thresholds in the two tests. For PTA in this study threshold levels were determined by increasing increments of 10 dB followed by decreasing increments of 5 dB, and for ASSR, measurements were performed using a descending procedure, by reducing the intensity of the acoustic signal in 10 dB steps until the threshold. The differences in step size and protocols used in PTA and ASSR can easily result in at least 5 dB difference in thresholds between the two tests. Additionally, this study used an automated KUDUwave audiometer to establish PTA and compared them to ASSR. There is a ± 5–10 dB difference between KUDUwave and clinician obtained (in an audiological clinical setting) hearing thresholds in frequencies between 1000 and 4000 Hz and more than 10 dB difference for 500 Hz (47). Therefore future research should compare ASSR thresholds with KUDUwave PTA and clinician obtained PTA.

In this study, a maximum threshold search time of 7 min was set for ASSR due to testing time constraints. However, in some participants no thresholds were establish during this 7 min time frame and ASSR thresholds were obtained at all four carrier frequencies for only 36% of the participant sample. For ASSR, it is unknown how much search time should be allowed for thresholds to be established, highlighting another ASSR protocol element that requires refining and further research to improve methodological quality and clinical application.

Furthermore, this study only included participants with normal hearing or mild hearing loss, which does not provide full insight into the use of ASSR for testing hearing acuity in older adults. Previous reports indicate that ASSR thresholds are closer to PTA thresholds in participants with sensorineural hearing loss in comparison to normal hearing participants (30, 36, 61). It has been suggested that such smaller threshold differences between ASSR and PTA in HL participants could be due to abnormal increase in the response amplitude as a result of recruitment for damage to outer hair cells (36, 61). Therefore, generalizing ASSR findings to normal hearing and HL groups could result in incorrect threshold estimation. Due to the limited sample size in this study and difficulties obtaining ASSR thresholds in some participants, exploring the reliability of ASSR threshold measures in participants based on hearing thresholds (those with normal hearing and those with mild HL) was not suitable. Future research would benefit from investigating ASSR threshold measures in older adults with different degrees of HL.

## Conclusion

The findings of the present study provide evidence to indicate that ASSR may be a valuable tool in estimating objective frequency-specific hearing thresholds in older adults. Though there is increased risk of false positive, due to over estimation of HL, ASSR is still reliable in assessing HL. Threshold differences between ASSR and the gold standard PTA were, for the majority of participants, within clinically acceptable ranges, thus ASSR can be useful in identifying HL in order to create hearing treatment plans for older adults who are unable to complete behavioral PTA. However, additional research is required to determine optimal parameters of ASSR for threshold estimation in older adults in order to increase its consistency and reliability, as well as eliminate some of the limitations associated with this technique. More research is also required to define modulation frequencies that are more suitable for older adults, which could provide valuable information to inform ASSR testing protocols for them as well as acquisition system manufacturers. Defining specific correction factors that take into account the patient's age, degree of HL and are specific for the carrier frequency can also help improve the methodological quality of ASSR.

## Author Contributions

HT, DJ, WM, HS, and RM conceived the idea for this study. HT collected hearing data with the supervision of DJ. HT prepared the initial draft with input from DJ, WM, and HS. All authors contributed to the development of the idea of this manuscript, as well as contributed to the revision of the manuscript. All authors approved for the manuscript to be submitted.

## Funding

This work was supported by the Australian Government Research Training Program Scholarship at The University of Western Australia; and Australian Alzheimer's Research Foundation.

## Conflict of Interest

The authors declare that the research was conducted in the absence of any commercial or financial relationships that could be construed as a potential conflict of interest.

## Publisher's Note

All claims expressed in this article are solely those of the authors and do not necessarily represent those of their affiliated organizations, or those of the publisher, the editors and the reviewers. Any product that may be evaluated in this article, or claim that may be made by its manufacturer, is not guaranteed or endorsed by the publisher.
