# Supplementary material for: Comparison of Auditory Steady-State Responses With Conventional Audiometry in Older Adults
Source: Front Neurol. 2022 Jul 4;13:924096. doi: 10.3389/fneur.2022.924096 (PMC9330634; doi:10.3389/fneur.2022.924096)
Supplement: Supplementary file 1 [file Data_Sheet_1.PDF]

Supplementary material

Table S1: Pearson correlation coefficient values (r) between PTA and ASSR results at each frequency for left and right ears.

Frequency	**Right Ear**		**Left Ear**		**Combined**	
	r Value	p Value	r Value	p Value	**r Value**	**p Value**
500 Hz	0.418	P 0.014	0.415	P 0.018	**0.418**	**p <0.001**
1000 Hz	0.596	P <0.001	0.473	P <0.001	**0.533**	**p <0.001**
2000 Hz	0.749	P <0.001	0.733	P <0.001	**0.737**	**p <0.001**
4000 Hz	0.836	P <0.001	0.846	P <0.001	**0.837**	**p <0.001**

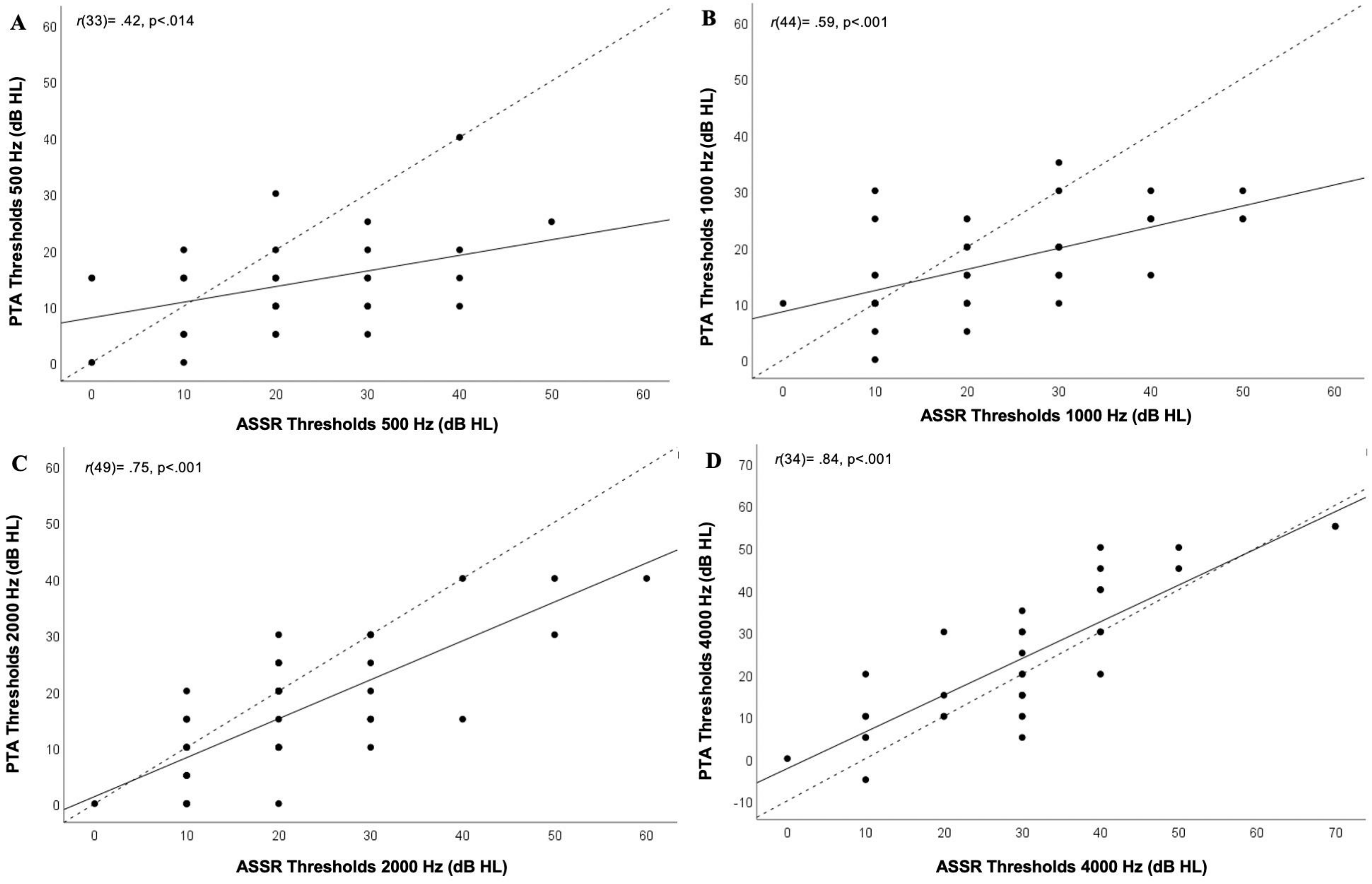

Figure S1: Correlation between hearing thresholds (Right ear only) obtained from auditory steady-state response (ASSR) (x-axis) and pure-tone audiometry (PTA) (y-axis) in dB HL according to carrier frequency. Correlation coefficients (*r*) and p-value are presented on the top left corner of each panel for all tested frequencies. Black line indicates line of best fit, dotted line indicates 1:1 ratio line.

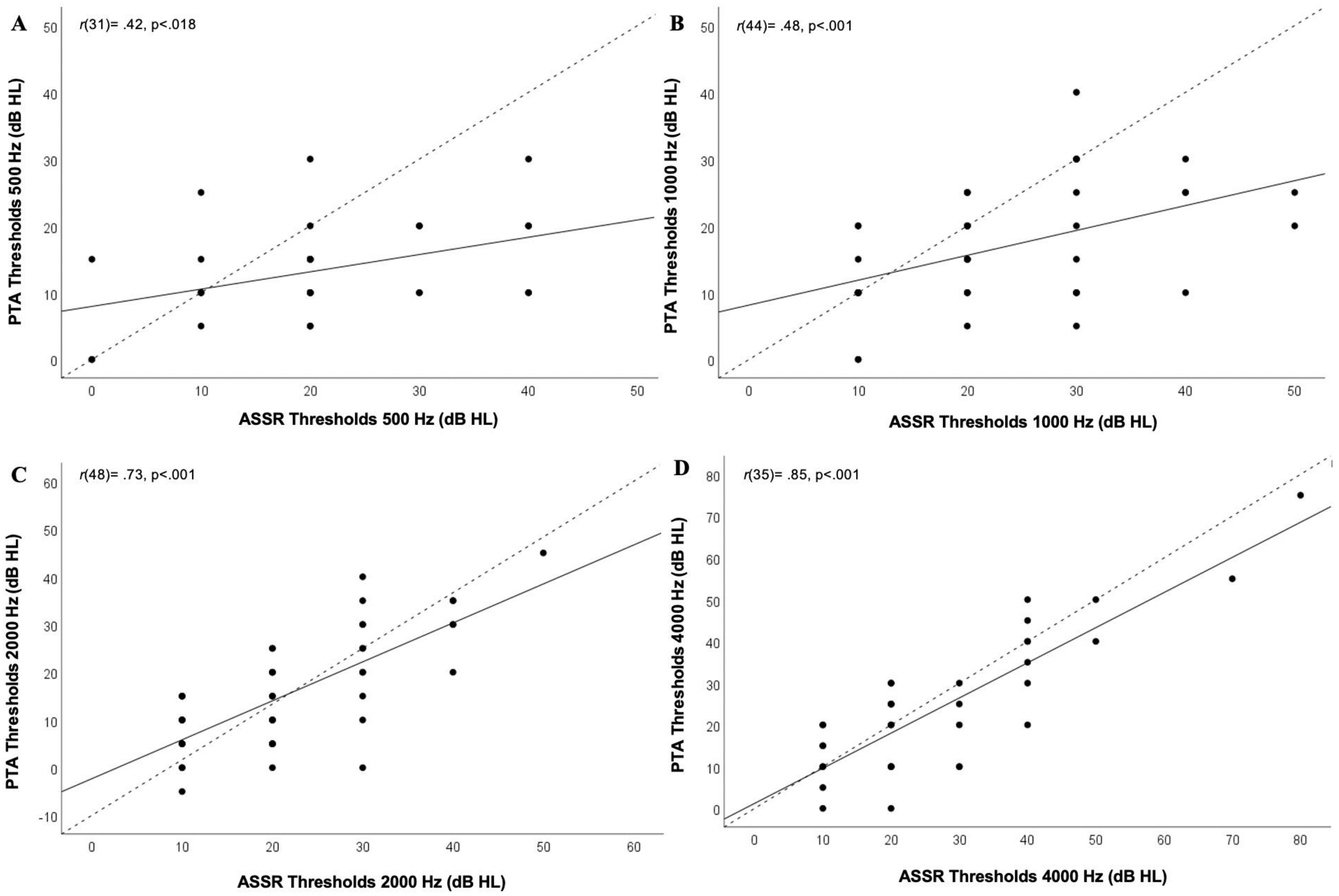

Figure S2: Correlation between hearing thresholds (Left ear only) obtained from auditory steady-state response (ASSR) (x-axis) and pure-tone audiometry (PTA) (y-axis) in dB HL according to carrier frequency. Correlation coefficients (r) and p-value are presented on the top left corner of each panel for all tested frequencies. Black line indicates line of best fit, dotted line indicates 1:1 ratio line.
